# Effects of User-Reported Risk Factors and Follow-Up Care Activities on Satisfaction With a COVID-19 Chatbot: Cross-Sectional Study

**DOI:** 10.2196/43105

**Published:** 2023-12-14

**Authors:** Akanksha Singh, Benjamin Schooley, Nitin Patel

**Affiliations:** 1 Department of Health Services Administration School of Health Professions University of Alabama at Birmingham Birmingham, AL United States; 2 IT & Cybersecurity Department of Electrical and Computer Engineering Brigham Young University Provo, UT United States; 3 Hackensack Meridian Health Hackensack, NJ United States

**Keywords:** patient engagement, chatbot, population health, health recommender systems, conversational recommender systems, design factors, COVID-19

## Abstract

**Background:**

The COVID-19 pandemic influenced many to consider methods to reduce human contact and ease the burden placed on health care workers. Conversational agents or chatbots are a set of technologies that may aid with these challenges. They may provide useful interactions for users, potentially reducing the health care worker burden while increasing user satisfaction. Research aims to understand these potential impacts of chatbots and conversational recommender systems and their associated design features.

**Objective:**

The objective of this study was to evaluate user perceptions of the helpfulness of an artificial intelligence chatbot that was offered free to the public in response to COVID-19. The chatbot engaged patients and provided educational information and the opportunity to report symptoms, understand personal risks, and receive referrals for care.

**Methods:**

A cross-sectional study design was used to analyze 82,222 chats collected from patients in South Carolina seeking services from the Prisma Health system. Chi-square tests and multinomial logistic regression analyses were conducted to assess the relationship between reported risk factors and perceived chat helpfulness using chats started between April 24, 2020, and April 21, 2022.

**Results:**

A total of 82,222 chat series were started with at least one question or response on record; 53,805 symptom checker questions with at least one COVID-19–related activity series were completed, with 5191 individuals clicking further to receive a virtual video visit and 2215 clicking further to make an appointment with a local physician. Patients who were aged >65 years (*P*<.001), reported comorbidities (*P*<.001), had been in contact with a person with COVID-19 in the last 14 days (*P*<.001), and responded to symptom checker questions that placed them at a higher risk of COVID-19 (*P*<.001) were 1.8 times more likely to report the chat as helpful than those who reported lower risk factors. Users who engaged with the chatbot to conduct a series of activities were more likely to find the chat helpful (*P*<.001), including seeking COVID-19 information (3.97-4.07 times), in-person appointments (2.46-1.99 times), telehealth appointments with a nearby provider (2.48-1.9 times), or vaccination (2.9-3.85 times) compared with those who did not perform any of these activities.

**Conclusions:**

Chatbots that are designed to target high-risk user groups and provide relevant actionable items may be perceived as a helpful approach to early contact with the health system for assessing communicable disease symptoms and follow-up care options at home before virtual or in-person contact with health care providers. The results identified and validated significant design factors for conversational recommender systems, including triangulating a high-risk target user population and providing relevant actionable items for users to choose from as part of user engagement.

## Introduction

### Background and Significance

Since the discovery of SARS-CoV-2 and subsequent COVID-19 disease outbreak began in December 2019, the pandemic has challenged health care systems and societies worldwide to respond in unprecedented ways to protect both patients and staff and prepare for surges of patients who are critically ill. Health care institutions have revamped services and delivery, pushing digital transformations and innovative models for crisis management and health care delivery at an unforeseen scale [1-3]. An important goal has been to manage patient load, directing a large number of patients and the general public to an appropriate level of care [4]. Indeed, a variety of patient support pathways for COVID-19 have become more commonplace [5], including those facilitated by telehealth, telephony [6], email, and interactive chat [7,8]. These technologies have been applied during the pandemic, for example, in primary care visits [9], patient-family communications [10], postdischarge follow-ups, and palliative care [11-13]. A range of benefits have been reported. For example, robust, physician-directed 24/7 COVID-19 response hotlines [14] can help meet increased health care service demands during the acute phase of a pandemic, conserving scarce resources such as personal protective equipment and testing supplies and preventing the spread of infections to patients and health care workers. These trends have motivated investigation into new and better ways to provide remote care facilitated by technology, such as remote patient monitoring, e-visits, e-consults, mobile health, and tele-education [15].

This study aimed to evaluate the impacts of an interactive conversational recommender telehealth system (ie, chatbot) for engaging users during the COVID-19 pandemic in the state of South Carolina to address the health care needs of users while also trying to reduce exposure to the virus for users and providers alike. We assessed the impact of its use to inform the design considerations of future conversational recommender systems.

During the period following the outbreak of COVID-19, health care delivery was dramatically pushed toward telehealth and telemedicine [16-19]. Telehealth technologies have provided an effective and efficient way to deliver health care during the pandemic [20,21] for older populations [22] and patients with chronic conditions [23] and to reduce burnout for physicians working under epidemic conditions [24]. Telehealth virtual visits have reduced in-person visits by 33% [25] and are believed to have significant potential for positive socioeconomic impact on health care delivery [26-28]. Telehealth has rapidly expanded to include low-acuity conditions (including acute respiratory infections) [29] and infectious disease consultation, diagnosis, and monitoring [30].

Virtual visits have taken different forms, including synchronous video visits either prescheduled or on demand [31], telephony-based care, and chat-based virtual visits with providers, and more recently, chatbots have been introduced [32,33]. Artificial intelligence (AI) chatbots have also made their way into remote care practice [34-38]. Although physicians have reported mixed views on the value of chatbots, some studies have found that providers acknowledge cost savings and benefits when the functionality of the chatbot plays a clear strategic role for an organization [39,40].

Since the COVID-19 outbreak, chatbots have been used to provide remote assessments to triage potential patients [41,42], expand access to health care education, and try to ease supply and demand challenges for human health care providers [43]. Challenges to wider adoption include the need for more humanlike conversations and social and ethical considerations [13,34,44,45].

### Chatbots for Patient Engagement and Satisfaction

Personalization and evaluation of chatbots are needed to enhance patient engagement and satisfaction. Direct-to-consumer telehealth satisfaction has generally been high among patients [46], with one study reporting satisfaction with 85% of virtual visits [47]. However, significant variation in quality has been found across virtual visit providers for the management of common acute illnesses [48]. In one study, the quality of telehealth was lacking in terms of appropriateness of ordering tests and scheduling follow-up visits for patients with acute respiratory infections [49].

Although satisfaction levels have been high for video-based direct-to-consumer virtual visits, little is known about satisfaction and patient engagement with emerging technologies such as asynchronous chatbots. Benefits have been found for the management of breast cancer [50] and the postoperative management of patients of orthopedics [51]. Other studies have reported low levels of patient engagement during ureteroscopic follow-up [52]. Factors affecting chatbot acceptance, encompassing engagement and satisfaction, include their perceived utility and trustworthiness. In contrast, factors such as poor patient computer skills and dislike for talking to computers have negatively affected patient satisfaction [32]. In contrast, the ability to personalize a chatbot experience, such as the selection of a preferred language, positively affects user trust and engagement [53]. Chatbots represent an interactive technology that requires assessment of its ability to engage users in a helpful and appealing way while reducing exposure and health care use.

### Patient Engagement During Pandemics

We found very limited research on patient engagement strategies during an epidemic using tools such as chatbots. A recent study reported on a self-triage and self-scheduling tool to stratify and recommend appropriate care for patients with COVID-19 [54]. A similar triage tool was recently implemented for patients with multiple sclerosis to identify those who are at high risk for the purpose of reducing COVID-19 spread at multiple sclerosis centers [55].

As with many new technologies, chatbots carry the inherent risks of possible user resistance and technology immaturity. Dependence on emergent and complex technologies such as AI and natural language processing represents a significant risk for health care organizations as AI and natural language processing are still far from providing humanlike experiences inclusive of human cognition and empathy [32,56]. In contrast, chatbots offer advantages and benefits, such as the possibility of simplifying information search processes, the availability of easy-to-use instant messaging–like user interfaces, the capability to leverage previous user interactions for sentiment analysis, and the ability to provide personalized responses [56], while also reducing health care provider workload [57]. Understanding the benefits and risks of these technologies as they mature is important for assessing their impacts and future directions. In this study, we assessed the user-perceived helpfulness of a chatbot implemented at a large health care system.

Satisfaction with technology is a key metric to assess the impact of patient engagement strategies and interventions such as the use of chatbots for providing telehealth services during the COVID-19 pandemic. Recent studies on advances in clinical recommender systems and chatbots point to a need to explore evaluation paradigms for chatbots [58,59], including necessary constructs such as user satisfaction and effectiveness [60,61]. In this study, we used these evaluation paradigms to identify factors that inform the design of chatbots targeted to increase user satisfaction and effectiveness. We analytically triangulated the target population for a COVID-19 chatbot designed to engage patients who are at risk or feel that they are at risk of the disease and correlated the reported risk factors with chat helpfulness. On the basis of guidance from the Centers for Disease Control and Prevention (CDC), users at higher risk include those who are aged ≥65 years, report cough or breathing difficulty in the past few weeks, report any of the CDC-specified relevant comorbidities, have been in close contact with anyone who has tested positive for COVID-19 within the past 14 days, report CDC-specified COVID-19 symptoms in the past week, and are identified by the health system as being in the high-risk symptom category. Thus, users who are at a higher risk of COVID-19 will report the chat to be helpful more frequently than the group of users who are at a lower risk of COVID-19 (hypothesis 1).

We propose that users who find a relevant actionable item to engage with during their chat session will report the chat to be more helpful. Engagement means that the user finds and clicks on a hyperlink that navigates them to the described item. There may be many ways to help users engage with an action item regarding COVID-19, such as directing them to a CDC-specified COVID-19 information page, relevant links or telephone numbers to seek care, in-person provider appointments in their geographic area, telehealth appointments, vaccination information, information and telephone numbers to seek vaccination appointments in their area, and CDC travel guidelines to help them prepare for potential exposure. We posit that the use of user-relevant actionable items during a chat session increases user reporting of chat helpfulness. Thus, we analytically identified the relevant actionable items and links that are associated with user satisfaction with the chatbot. Hence, users who engage with an actionable item during their chat session will report the chat to be more helpful (hypothesis 2).

## Methods

### Overview

A case study approach was used to analyze a chatbot implementation by a large health system in the Southeastern United States. The health system aimed to provide free chatbot access to COVID-19 information resources, symptom checking, and referrals for additional in-person or virtual care to the public. Chatbot responses were collected by the case study organization and then analyzed by researchers using chi-square tests and multinomial logistic regression models to find significant differences between groups in which categorical responses were provided.

### Study Setting

Prisma Health is the largest and most comprehensive health care system in the state of South Carolina, treating 1.2 million unique patients in the 2019 financial year with 330 physician practice sites and 18 hospitals inclusive of 2984 beds, 30,000 team members, 2 level-1 trauma centers, 2 comprehensive stroke centers, 2 affiliated medical schools and nursing schools, 50 residency and fellowship programs, and 560 residents or fellows. The health system serves a geographic area where 45% of South Carolina’s 5.2 million residents live within a 15-minute drive [62]. As with most health care organizations grappling with COVID-19, Prisma Health was challenged with how best to provide access to care while protecting patients and employees from infection during the pandemic. There are significant population health challenges in South Carolina, including a high prevalence of chronic conditions. South Carolina was ranked 42 of all US states in terms of health determinants and outcomes in 2019 [63]. To address the needs of their patient population, Prisma Health developed a strategy for virtual care to serve as an important entry point into the health care system for both existing patients and the inquiring public. Easy-to-find connection points were constructed between asynchronous and synchronous virtual visits and scheduling of in-person primary care and specialty care services.

### Chatbot Pathway

In response to COVID-19, Prisma Health moved its multiyear strategy to rapidly implement a chatbot in early 2020. Telehealth offerings were expanded to include virtual primary care visits, a free COVID-19 telehealth screening service, and a 1-800 telephony-based COVID-19 hotline. In April 2020, a chatbot was implemented to provide COVID-19 education; symptom screening; and referrals for follow-up care to the telephony service, video telehealth screening, and physicians’ offices across the Prisma Health system. Chatbot technology is an essential component of Prisma Health’s overall virtual care strategy to engage users with initial questions, guide users to appropriate levels of care, and provide a helpful experience to users while reducing staff time involved in discussing issues that can be more effectively addressed with interactive technologies. The chatbot asked a range of questions to individuals who freely and voluntarily used the system. The range and types of questions asked depended on how each individual user responded to the previous chatbot question, formulated as a decision tree. The chatbot placed each user in a symptom risk zone based on their responses. For the symptom zone assessment, red-yellow-green categories were predetermined by a clinician committee. “Red” was assigned to a chatbot user who reported any symptom or combination of symptoms with or without any other reported risk factor. For example, a person reporting “fever” (question 7) and a comorbidity of diabetes (question 4) was assigned the “Red” risk level. “Yellow” was assigned to any chatbot user reporting any comorbidity or close contact with a patient with COVID-19 in the last 14 days or who reported being aged >65 years. For example, a person not reporting any symptoms (question 7) but having a comorbidity (question 4) of diabetes was assigned “Yellow.” “Green” was assigned to users who did not report risk factors. The effective start date for the symptom zone assessment was September 16, 2020.

**Figure 1:**
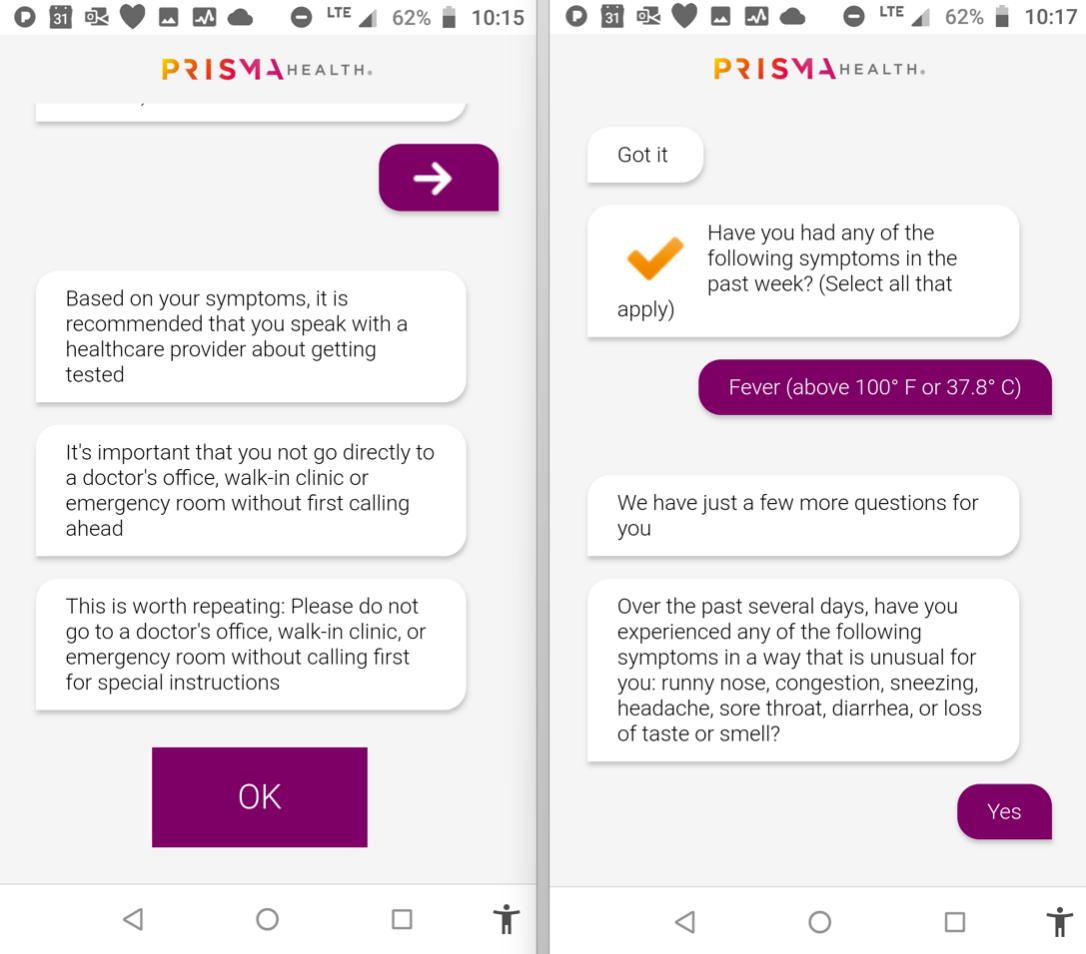
Screenshot of a partial Prisma Health chatbot COVID-19 symptom checker conversation.

Figure 1 shows screenshots of a partial chatbot conversation checking for COVID-19 symptoms in a patient. As shown in these figures, relevant instructions or suggestions were provided to the user following each patient interaction with the symptom checker. Assessment of the Prisma Health COVID-19 chatbot for its helpfulness to consumers was core and central to this study. As noted previously, it was believed that the chatbot would be viewed as helpful by those who reported risk factors associated with the disease compared with those who reported no or fewer risk factors.

### Data Collection and Analysis

#### Data Collection and Coding

We collected data from the Prisma Health chatbot (powered by Conversa) between April 24, 2020, and April 21, 2022. Data were received including user chatbot responses and user follow-up actions (ie, activities). Secondary analysis of data included analysis of individual chat responses to COVID-19 symptom questions; education resources that were consumed about COVID-19; and user activities pertaining to seeking an in-person or telehealth appointment, seeking a COVID-19 vaccination, and responses to a question regarding the helpfulness of the chat.

We used Python with the Pandas library (Python Software Foundation) to transform data from chat user symptom responses and chat user action responses to create one response and activity series for each user in each row. We then coded each response variable (ie, question 1, question 2, and so on). We coded all missing responses as “SYSMIS” and excluded them from the analysis. “Yes” and “No” responses were coded as 0 and 1, respectively. Questions that included “No,” “Somewhat,” and “Yes” responses were coded as 0, 0.5, and 1, respectively (eg, question 2). Question 6 was coded as “No”=0, “Yes”=1, and “Not sure”=2. Symptoms in question 7 were divided into 4 categories: “No symptom” (coded as 0), “1 symptom” (coded as 1), “2 symptoms” (coded as 2), and “3 symptoms” (coded as 3). Similarly, symptom zone assignment by the chatbot in question 12 was classified as “Green” (0), “Yellow” (1), and “Red” (2).

User activities were coded into 7 categories: “Seeking COVID-19 information” (A1), “Contact Prisma Health” (A2), “Seeking in-person appointment” (A3), “Seeking telehealth appointment” (A4), “Seeking vaccination” (A5), “Seeking travel guidelines” (A6), and “Seeking vaccination information” (A7). Multimedia Appendix 1 illustrates how coded action categories (eg, seeking COVID-19 information) were mapped to the actual hyperlinks located in the chatbot. Any user who recorded one or more activities in a chat session was coded as 1, whereas users who did not engage in a chatbot activity were coded as 0. Each of the activity items was represented in the chatbot as internal Prisma Health hyperlinks or external hyperlinks for users to click to engage in actions outside the chatbot, including seeking additional information, calling a phone number, finding local physicians, or making a web-based appointment.

#### Data Analysis

We analyzed the data using the SPSS Statistics software (version 27; IBM Corp). First, we analyzed the frequencies and proportions of categorical variables to describe the characteristics of the participants and describe user responses and activities. As all the variables being analyzed were categorical, missing data for chat responses were labeled as missing (SYSMIS) and excluded from the analysis rather than applying data imputation. Second, we conducted chi-square tests to compare the differences in the distribution of risk factor responses and chat helpfulness. Third, we conducted multinomial logistic regression to estimate the odds ratios (ORs) with 95% CIs of various categorical responses for several COVID-19 risk factors and activity variables (independent variables) on the categorical dependent variable (ie, chat helpfulness). Chatbot user responses including “Yes” or “Somewhat” were controlled to examine the robustness of the associations. We used the multinomial logistic regression method for OR analysis as it is helpful in determining associations between nominal (categorical) variables that are not ordinal [64]. In the basic model, we examined the association between chat helpfulness and symptom zone. In model 1, we included the responses to the question on age-related risk. In model 2, we included the responses to the remaining risk questions. While analyzing the impact of user-selected activities on chat helpfulness, we used a basic model to estimate the ORs with 95% CIs of various activity groups that were found to significantly affect the chat helpfulness responses in step 3.

We further explored models to enhance the basic model for chat helpfulness and activities by adding responses as covariates. We stopped when there were insufficient data to establish any further improvement in significance and OR.

### Ethics Approval

This study was conducted under the University of South Carolina institutional review board–approved protocol (Pro00101062).

## Results

### Overview

Between April 24, 2020, and April 21, 2022, a total of 82,222 chat series were started with at least one question or response on record. A total of 53,805 symptom checker questions were accompanied by at least one COVID-19–related activity by users. Among those activities, 5191 chat users clicked further to receive a virtual video visit, and 2215 clicked to make an appointment with a local physician. Chatbot users reported using the tool primarily for the following reasons: checking symptoms, learning about the virus, learning about treatment, learning how to avoid infection, signing up for SMS text message alerts, or accomplishing something else. A total of 9931 users answered the following question: “Did you find this chat helpful?” Similarly structured and phrased questions and response choices about app [65], provider [66-68], and health care program helpfulness [69] have been used in previous research. Chat participants were unique users defined by email addresses, and their responses were analyzed further. Table 1 shows the frequency distributions of user responses to the chatbot questions.

**Table 1:** Chatbot response and activity frequencies (N=53,805).

COVID-19 risk question or user activity and response classification and coding of categories		Response	Total response series, n (%)
**Are you aged ≥65 years? (question 1)**			
	0	No (0)	43,135 (80.17)
	1	Yes (1)	3817 (7.09)
	SYSMIS	Missing (system)	6853 (12.74)
**Did you find this chat helpful? (question 2)**			
	0	No (0)	3346 (6.22)
	1	Yes (1)	3971 (7.38)
	0.5	Somewhat (0.5)	2614 (4.86)
	SYSMIS	Missing (system)	43,874 (81.54)
**Did your cough or breathing difficulty start or become significantly worse sometime in the past few weeks? (question 3)**			
	0	No (0)	9113 (16.94)
	1	Yes (1)	16,922 (31.45)
	SYSMIS	Missing (system)	27,770 (51.61)
**Do you have any of the following conditions: diabetes, heart disease, chronic lung disease, or any condition that lowers your body’s ability to fight infection (pregnancy or chemotherapy or steroids for cancer or sickle cell disease)? (question 4)**			
	0	No (0)	37,934 (70.5)
	1	Yes (1)	8805 (16.36)
	SYSMIS	Missing (system)	7066 (13.13)
**Have you been in close contact with anyone who has tested positive for COVID-19 within the past 14 days? (question 6)**			
	0	No (0)	10,986 (20.42)
	2	Not sure (2)	22,061 (41)
	1	Yes (1)	13,584 (25.25)
	SYSMIS	Missing (system)	7174 (13.3)
**Have you had any of the following symptoms in the past week? (select all that apply; question 7)**			
	No symptom (0)	None of these (0)	18,570 (34.51)
	1 symptom (1)	Cough (1)	13,328 (24.77)
	1 symptom (1)	Shortness of breath (1)	3357 (6.24)
	1 symptom (1)	Fever (>100 °F or >37.8 °C; 1)	4088 (7.6)
	2 symptoms (2)	Fever (>100 °F or >37.8 °C); cough (2)	2345 (4.36)
	2 symptoms (2)	Fever (>100 °F or >37.8 °C); shortness of breath (2)	427 (0.79)
	2 symptoms (2)	Cough; shortness of breath (2)	4833 (8.98)
	3 symptoms (3)	Fever (>100 °F or >37.8 °C); cough; shortness of breath (3)	1490 (2.77)
	SYSMIS	Missing (system)	5367 (9.97)
**Over the past several days, have you experienced any of the following symptoms in a way that is unusual for you: runny nose, congestion, sneezing, headache, sore throat, diarrhea, or loss of taste or smell? (question 11)**			
	0	No (0)	10,408 (19.34)
	1	Yes (1)	36,772 (68.34)
	SYSMIS	Missing (system)	6625 (12.31)
**Symptom zone (system assigned; question 12)**			
	Green (0)	Green (0)	2493 (4.63)
	Yellow (1)	Yellow or yellow plus (1)	15,519 (28.84)
	Red (2)	Red (2)	13,601 (25.28)
	SYSMIS	Missing (system)	22,192 (41.25)
**Seeking COVID-19 information (A1^a^)**			
	1	Seeking COVID-19 information	2718 (5.05)
	0	Missing (system)	51,087 (94.95)
**Contact Prisma Health (A2)**			
	1	Contact Prisma Health	2 (0)
	0	Missing (system)	53,803 (100)
**Seeking in-person appointment (A3)**			
	1	Seeking in-person appointment	2215 (4.12)
	0	Missing (system)	51,590 (95.88)
**Seeking telehealth appointment (A4)**			
	1	Seeking telehealth appointment	5191 (9.65)
	0	Missing (system)	48,614 (90.35)
**Seeking vaccination (A5)**			
	1	Seeking vaccination	4477 (8.32)
	0	Missing (system)	49,328 (91.68)
**Seeking travel guidelines (A6)**			
	1	Seeking travel guidelines	29 (0.05)
	0	Missing (system)	53,776 (99.95)
**Seeking vaccination information (A7)**			
	1	Seeking vaccination information	3177 (5.9)
	0	Missing (system)	50,628 (94.1)

^a^A1-A7: user-selected activities.

### Significant COVID-19 Risk Factors

**Figure 2:**
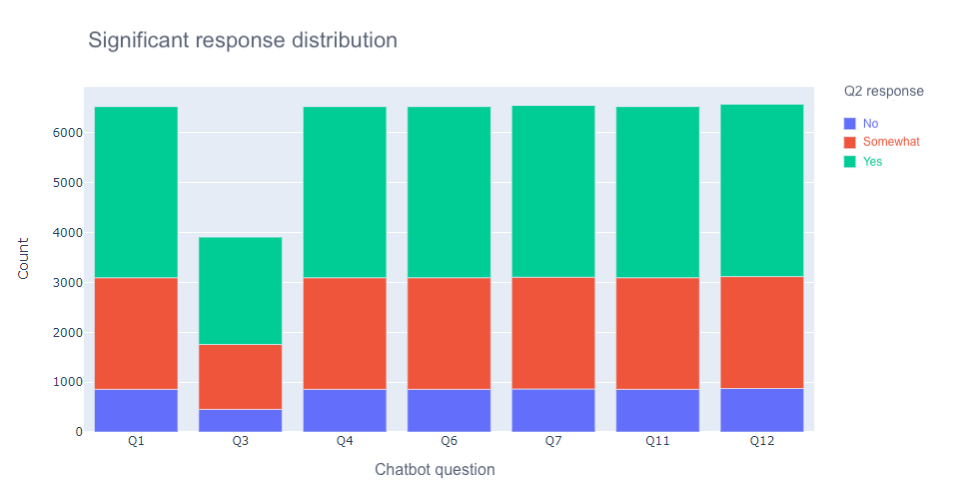
Frequency of chat helpfulness responses as they correlate with significant COVID-19 risk assessment questions. Q1: question 1; Q2: question 2; Q3: question 3; Q4: question 4; Q6: question 6; Q7: question 7; Q11: question 11; Q12: question 12.

The Prisma Health Conversa chatbot decision tree uses the selections from a user to determine the symptom zone. User responses to COVID-19 risk factors such as age of >65 years in question 1, cough and breathing difficulty in question 3, comorbidities in question 4, close contact with a person with COVID-19 in question 6, symptom list in question 7, and additional recent general symptoms in question 11 were calculated to determine a symptom zone in question 12 for each series of questions answered. Figure 2 represents the frequency distributions of chat helpfulness responses in relation to these risk factor questions. Table 2 displays the results of the chi-square tests. Each symptom question (1, 3, 4, 6, 7, 11, and 12) was individually assessed for significance in determining chat helpfulness (Table 2). Further analyses using multinomial logistic regression are presented in the following section.

**Table 2:** Chatbot user risk factor questionnaire responses and COVID-19–related activity characteristics in various chat helpfulness response groups.

COVID-19 risk question or user activity and response category			Responses to “Did you find this chat helpful?” (question 2), n (%)						Pearson chi-square *P* value	
			No		Somewhat		Yes			
**Are you aged ≥65 years? (question 1)**										<.001
	No (n=5820)	799 (13.7)		2025 (34.8)		2996 (51.5)				
	Yes (n=708)	58 (8.2)		214 (30.2)		436 (61.6)				
	Total (n=6528)	857 (13.1)		2239 (34.3)		3432 (52.6)				
**Did your cough or breathing difficulty start or become significantly worse sometime in the past few weeks? (question 3)**										<.001
	No (n=1141)	176 (15.4)		415 (36.4)		550 (48.2)				
	Yes (n=2770)	279 (10.1)		887 (32)		1604 (57.9)				
	Total (n=3911)	455 (11.6)		1302 (33.3)		2154 (55.1)				
**Do you have any of the following conditions: diabetes, heart disease, chronic lung disease, or any condition that lowers your body’s ability to fight infection (pregnancy or chemotherapy or steroids for cancer or sickle cell disease)? (question 4)**										<.001
	No (n=4994)	697 (14)		1722 (34.5)		2575 (51.6)				
	Yes (n=1534)	160 (10.4)		517 (33.7)		857 (55.9)				
	Total (n=6528)	857 (13.1)		2239 (34.3)		3432 (52.6)				
**Have you been in close contact with anyone who has tested positive for COVID-19 within the past 14 days? (question 6)**										<.001
	No (n=1519)	183 (12.1)		441 (29)		895 (58.9)				
	Yes (n=1789)	317 (17.7)		598 (33.4)		874 (48.9)				
	Not sure (n=3222)	357 (11.1)		1200 (37.2)		1665 (51.7)				
	Total (n=6530)	857 (13.1)		2239 (34.3)		3434 (52.6)				
**Have you had any of the following symptoms in the past week? (select all that apply; question 7)**										<.001
	No symptom (n=2180)	359 (16.5)		772 (35.4)		1049 (48.1)				
	1 symptom (n=2587)	293 (11.3)		906 (35)		1388 (53.7)				
	2 symptoms (n=1516)	181 (11.9)		479 (31.6)		856 (56.5)				
	3 symptoms (n=269)	30 (11.2)		87 (32.3)		152 (56.5)				
	Total (n=6552)	863 (13.2)		2244 (34.3)		3445 (52.6)				
**Over the past several days, have you experienced any of the following symptoms in a way that is unusual for you: runny nose, congestion, sneezing, headache, sore throat, diarrhea, or loss of taste or smell? (question 11)**										.001
	No (n=1301)	184 (14.1)		391 (30)		726 (55.8)				
	Yes (n=5226)	673 (12.9)		1848 (35.4)		2705 (51.8)				
	Total (n=6527)	857 (13.1)		2239 (34.3)		3431 (52.6)				
**Symptom zone (system assigned; question 12)**										<.001
	Green (n=440)	82 (18.6)		110 (25)		248 (56.4)				
	Yellow (n=2781)	454 (16.3)		1034 (37.2)		1293 (46.5)				
	Red (n=3356)	337 (10)		1102 (32.8)		1917 (57.1)				
	Total (n=6577)	873 (13.3)		2246 (34.1)		3458 (52.6)				
**Seeking COVID-19 information (A1^a^)**										<.001
	Yes (n=338)	39 (11.5)		117 (34.6)		182 (53.8)				
**Contact Prisma Health (A2)**										N/A^b^
	Yes (n=0)	0 (0)		0 (0)		0 (0)				
**Seeking in-person appointment (A3)**										<.001
	Yes (n=188)	36 (19.1)		68 (36.2)		84 (44.7)				
**Seeking telehealth appointment (A4)**										<.001
	Yes (n=246)	48 (19.5)		91 (37)		107 (43.5)				
**Seeking vaccination (A5)**										<.001
	Yes (n=502)	67 (13.3)		146 (29.1)		289 (57.6)				
**Seeking travel guidelines (A6)**										.96
	Yes (n=3)	1 (33.3)		1 (33.3)		1 (33.3)				
**Seeking vaccination information (A7)**										.87
	Yes (n=656)	225 (34.3)		167 (25.5)		264 (40.2)				

^a^A1-A7: user-selected activities.

^b^N/A: not applicable; no statistics could be computed.

### Significant User Activities

**Figure 3:**
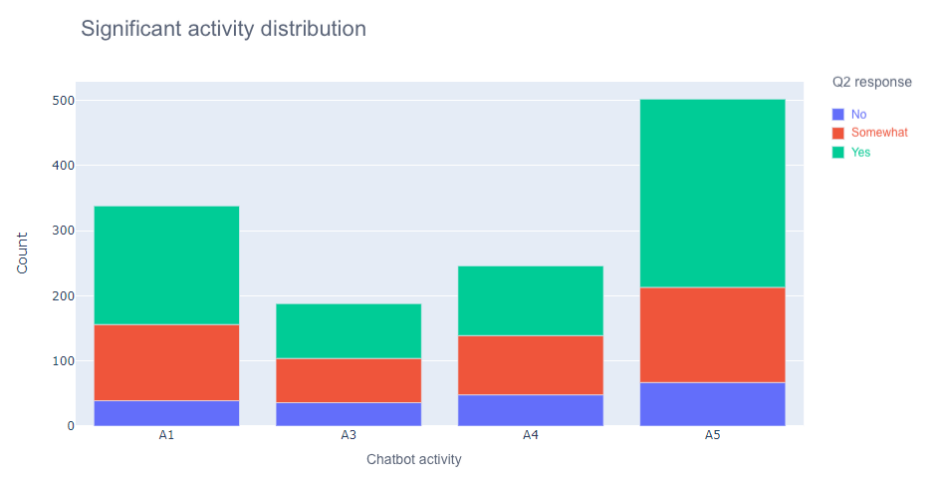
Frequency of chat helpfulness responses in relation to significant chatbot-prompted activities. Q2: question 2.

We conducted chi-square tests to determine the significance of various user-selected activities in relation to chat helpfulness (Table 2). Activities such as seeking travel guidelines and vaccination-related educational information were not found to be significant factors in determining chat helpfulness. Statistically significant relationships were found (*P*<.001) between chat helpfulness and several activities, including seeking COVID-19 information (A1), in-person appointments (A3), telehealth appointments (A4), and vaccination (A5). Figure 3 represents the frequency distributions of chat helpfulness responses in relation to chatbot-prompted activities that were found to be significant. Additional multinomial logistic regression analyses are reported in the following section.

### Relationship Between Persons at High Risk of COVID-19 and Positive Chat Helpfulness

To analyze the effect of chat responses and COVID-19 risk factors on chat helpfulness, we applied a multinomial logistic regression (Table 3). To test hypothesis 1, we examined the relationship between chat helpfulness and COVID-19 risk factor symptom zone. We tested 3 models, including a basic model 0, and then subsequent models in which we adjusted for age risk factor (question 1; model 1) and then for all risk factors (questions 1, 3, 4, 6, 7, and 11; model 2).

**Table 3:** Multinomial logistic regression of chat helpfulness for COVID-19 risk factor variables.

Question (variable) and response category				Odds ratio (95% CI)^a^				
				Basic model^b^		Model 1^c^		Model 2^d^
**Chat “somewhat” helpful (dependent category)**								
	**Symptom zone (question 12)**							
		Green	0.410 (0.301-0.560)^e^		0.471 (0.341-0.651)^e^		0.339 (0.123-0.938)^f^	
		Yellow	0.696 (0.591-0.821)^e^		0.674 (0.571-0.795)^e^		0.550 (0.345-0.878)^f^	
		Red	Reference		Reference		Reference	
	**Are you aged ≥65 years? (question 1)**							
		No	N/A^g^		0.668 (0.493-0.906)^h^		0.877 (0.562-1.368)	
		Yes	N/A		Reference		Reference	
	**Have you had any of the following symptoms in the past week? (select all that apply; question 3)**							
		1 symptom	N/A		N/A		1.257 (0.788-2.006)	
		2 symptoms	N/A		N/A		0.948 (0.599-1.501)	
		3 symptoms	N/A		N/A		Reference	
	**Did your cough or breathing difficulty start or become significantly worse sometime in the past few weeks? (question 4)**							
		No	N/A		N/A		1.097 (0.717-1.677)	
		Yes	N/A		N/A		Reference	
	**Over the past several days, have you experienced any of the following symptoms in a way that is unusual for you: runny nose, congestion, sneezing, headache, sore throat, diarrhea, or loss of taste or smell? (question 6)**							
		No	N/A		N/A		1.166 (0.770-1.764)	
		Yes	N/A		N/A		Reference	
	**Do you have any of the following conditions: diabetes, heart disease, chronic lung disease, or any condition that lowers your body’s ability to fight infection (pregnancy or chemotherapy or steroids for cancer or sickle cell disease)? (question 7)**							
		No	N/A		N/A		0.881 (0.676-1.148)	
		Yes	N/A		N/A		Reference	
	**Have you been in close contact with anyone who has tested positive for COVID-19 within the past 14 days? (question 11)**							
		No	N/A		N/A		1.135 (0.820-1.571)	
		Yes	N/A		N/A		0.569 (0.448-0.723)^e^	
		Not sure	N/A		N/A		Reference	
“**Yes” chat helpful (dependent category)**								
	**Symptom zone (question 12)**							
		Green	0.532 (0.404-0.700)^e^		0.606 (0.453-0.810)^e^		0.555 (0.218-1.409)	
		Yellow	0.501 (0.428-0.586)^e^		0.471 (0.402-0.553)^e^		0.416 (0.266-0.650)^e^	
		Red	Reference		Reference		Reference	
	**Are you aged ≥65 years? (question 1)**							
		No	N/A		0.445 (0.333-0.593)^e^		0.680 (0.446-1.037)	
		Yes	N/A		Reference		Reference	
	**Have you had any of the following symptoms in the past week? (select all that apply; question 3)**							
		1 symptom	N/A		N/A		1.208 (0.776-1.880)	
		2 symptoms	N/A		N/A		0.993 (0.643-1.533)	
		3 symptoms	N/A		N/A		Reference	
	**Did your cough or breathing difficulty start or become significantly worse sometime in the past few weeks? (question 4)**							
		No	N/A		N/A		0.929 (0.619-1.394)	
		Yes	N/A		N/A		Reference	
	**Over the past several days, have you experienced any of the following symptoms in a way that is unusual for you: runny nose, congestion, sneezing, headache, sore throat, diarrhea, or loss of taste or smell? (question 6)**							
		No	N/A		N/A		1.301 (0.877-1.931)	
		Yes	N/A		N/A		Reference	
	**Do you have any of the following conditions: diabetes, heart disease, chronic lung disease, or any condition that lowers your body’s ability to fight infection (pregnancy or chemotherapy or steroids for cancer or sickle cell disease)? (question 7)**							
		No	N/A		N/A		0.780 (0.606-1.003)	
		Yes	N/A		N/A		Reference	
	**Have you been in close contact with anyone who has tested positive for COVID-19 within the past 14 days? (question 11)**							
		No	N/A		N/A		1.342 (0.984-1.832)	
		Yes	N/A		N/A		0.591 (0.471-0.742)^e^	
		Not sure	N/A		N/A		Reference	

^a^The odds ratios and 95% CIs for the “Somewhat” and “Yes” responses for chat helpfulness were calculated, with “No” as the reference sample.

^b^In the basic model, we examined the association between symptom zone (question 12) and chat helpfulness (question 2).

^c^In model 1, we also adjusted for COVID-19 age risk factor (question 1) over the basic model.

^d^In model 2, we also adjusted for all COVID-19 risk factors (questions 1, 3, 4, 6, 7, and 11) over the basic model.

^e^*P*<.001.

^f^*P*<.05.

^g^N/A: not applicable; this variable was adjusted in subsequent models.

^h^*P*<.01.

From the 2 years of data on COVID-19 chatbot use, in 18.46% (9931/53,805) of chats, the question regarding chat helpfulness (question 2) was answered. In 26.32% (2614/9931) of these chats, the chat was reported to be “Somewhat” helpful, and in 39.99% (3971/9931), the chat was reported to be helpful (“Yes”; Table 1). Of the 6585 chats in which chat helpfulness was reported, 440 (6.68%) were assigned the “Green” symptom zone, 2781 (42.23%) were assigned the “Yellow” symptom zone, and 3356 (50.96%) were assigned the “Red” symptom zone.

As noted in Table 3, we found that the chat series that were assigned the “Green” symptom zone were 0.410 (95% CI 0.301-0.560) times less likely to find the chat somewhat helpful compared with the reference category of “Red” symptom zone. Similarly, chat series that were assigned the “Yellow” symptom zone were 0.696 (95% CI 0.591-0.821) times less likely to find the chat somewhat helpful compared with the reference category of “Red” symptom zone.

When adjusted for the age risk variable (question 1) in *model 1*, the adjusted OR (aOR) of finding the chat somewhat helpful slightly decreased in the “Yellow” symptom zone (aOR 0.674, 95% CI 0.571-0.795) compared with the “Red” symptom zone (reference category) but slightly increased for the “Green” symptom zone (aOR 0.471, 95% CI 0.341-0.651) compared with the “Red” symptom zone. In *model 2*, when adjusted over model 1 for the rest of the risk assessment variables (questions 3, 4, 6, 7, and 11), the aORs of both the “Green” (aOR 0.339, 95% CI 0.123-0.938) and “Yellow” (aOR 0.550, 95% CI 0.345-0.878) symptom zones decreased significantly compared with that of the “Red” symptom zone.

In summary, the chat series that were assigned a “Red” (higher) symptom zone were approximately 1.8 times more likely to find the chat somewhat helpful compared with the chat series assigned a “Yellow” symptom zone and approximately 2.95 times more likely to find the chat somewhat helpful compared with chat series assigned a “Green” (lower) symptom zone. Thus, we conclude that the chat series that were assigned a higher risk of COVID-19 were more likely to find the chat somewhat helpful compared with those that were assigned a lower COVID-19 risk level. The OR increased when adjusted for age and other risk categories.

Similarly, in the basic model, chat series that were assigned a “Green” symptom zone were 0.532 times (95% CI 0.404-0.700) less likely to answer “Yes” than those assigned a “Red” symptom zone. Those with a “Yellow” symptom zone were 0.501 times (95% CI 0.428-0.586) less likely to answer “Yes” than those assigned a “Red” symptom zone. When adjusted for the age risk factor (question 1) in model 1, the likelihood of the chat series in the “Green” symptom zone answering “yes” slightly increased (aOR 0.606, 95% CI 0.453-0.810) compared with chat series in the “Red” symptom zone but slightly decreased for chat series in the “Yellow” symptom zone (aOR 0.471, 95% CI 0.402-0.553). In model 2, when adjusted for the remaining risk factors (questions 3, 4, 6, 7, and 11), the aOR of the “Yellow” symptom zone (aOR 0.416, 95% CI 0.266-0.650) decreased compared with that of the basic model, whereas for the “Green” symptom zone (aOR 0.555, 95% CI 0.218-1.409), the aOR increased slightly when compared with that of the basic model.

To summarize, after adjusting for all COVID-19 risk factor questions in model 2, the chat series that were assigned the “Red” symptom zone were approximately 1.8 times more likely to respond to the chat helpfulness question (question 2) with a “Yes” compared with the chat series that were assigned the “Green” symptom zone. Similarly, the chat series that were assigned the “Red” symptom zone were approximately 2.4 times more likely to answer the chat helpfulness question (question 2) with a “Yes” compared with the chat series that were assigned the “Yellow” symptom zone.

To conclude, higher COVID-19 risk associations in the chat series resulted in a greater likelihood of positive chat helpfulness from users. The results indicate that people in higher COVID-19 risk categories may find the chat more positively helpful than those in lower COVID-19 risk categories, supporting hypothesis 1.

### Relationship Between Chat User Activity and Positive Chat Helpfulness

We further analyzed chat activities in association with chat helpfulness using multinomial regression (Table 4). As the activities were mutually exclusive for each chat response, we did not analyze the interassociations of activities with each other. Thus, we used a basic model to determine the ORs of each relevant activity and chat helpfulness.

**Table 4:** Multinomial logistic regression for chat helpfulness and activities.

Chat helpfulness question and response category		Odds ratio (95% CI)^a^	
		Chat “somewhat” helpful	“Yes” chat helpful
**Seeking COVID-19 information^b^ (A1^c^)**			
	No (0)	0.252 (0.175-0.363)^d^	0.246 (0.173-0.348)^d^
	Yes (1)	Reference	Reference
**Seeking in-person appointment^e^ (A3)**			
	No (0)	0.407 (0.271-0.612)^d^	0.503 (0.340-0.746)^d^
	Yes (1)	Reference	Reference
**Seeking telehealth appointment^f^ (A4)**			
	No (0)	0.404 (0.283-0.575)^c^	0.526 (0.373-0.741)^c^
	Yes (1)	Reference	Reference
**Seeking vaccination^g^ (A5)**			
	No (0)	0.345 (0.257-0.463)^c^	0.260 (0.199-0.341)^c^
	Yes (1)	Reference	Reference

^a^The odds ratios and 95% CIs for the “Somewhat” and “Yes” responses for chat helpfulness were calculated, with “No” as the reference sample.

^b^In this category, we examined the association between seeking COVID-19 information and chat helpfulness (question 2).

^c^A1-A7: user-selected activities.

^d^*P*<.001.

^e^In this category, we examined the association between seeking an in-person appointment and chat helpfulness (question 2).

^f^In this category, we examined the association between seeking a telehealth appointment and chat helpfulness (question 2).

^g^In this category, we examined the association between seeking vaccination and chat helpfulness (question 2).

In the 9931 chat series in which users answered the chat helpfulness question (question 2), some users carried out a variety of activities after selecting symptoms and receiving a symptom zone assignment. The activities with significant associations with positive chat helpfulness responses included seeking COVID-19 information (A1; 338/9931, 3.4%); seeking in-person appointments with Prisma Health network general practitioners (A3; 188/9931, 1.89%); seeking telehealth services (A4; 246/9931, 2.48%); and, finally, seeking vaccination (A5; 502/9931, 5.05%).

When analyzing these activities for association with chat helpfulness, we compared the ORs of the “Somewhat” and “Yes” chat helpfulness categories with the “No” chat helpfulness response as a reference category. Table 4 presents the ORs of the chat series for these activities, with the type of activity as the reference category. For this analysis, the chat series that sought these activities were in reference categories; hence, the ORs were inverted, and the β values were negative.

The user chat series that did not seek COVID-19 information were 0.252 times (95% CI 0.175-0.363) less likely to find the chat somewhat helpful and 0.246 times (95% CI 0.173-0.348) less likely to respond to the chat helpfulness question (question 2) with a “Yes” (reference category) than those that sought COVID-19 information. The user chat series that did not seek in-person appointments were 0.407 times (95% CI 0.271-0.612) less likely to find the chat somewhat helpful and 0.503 times (95% CI 0.340-0.746) less likely to respond to the chat helpfulness question (question 2) with a “Yes” than those that sought in-person appointments. The user chat series that did not seek telehealth appointments were 0.404 times (95% CI 0.283-0.575) less likely to find the chat somewhat helpful and 0.526 times (95% CI 0.373-0.741) less likely to respond to the chat helpfulness question (question 2) with a “Yes” than those that sought a telehealth appointment. Finally, user chat series that did not seek vaccination were 0.345 times (95% CI 0.257-0.463) less likely to find the chat somewhat helpful and 0.260 times (95% CI 0.199-0.341) less likely to respond to the chat helpfulness question (question 2) with a “Yes” than those that sought vaccination.

In summary, the chat series that sought additional follow-up activities (A1, A3, A4, and A5) found the chat to be more positively helpful than those that did not seek these activities. Users seeking COVID-19 information were approximately 3.97 times more likely to find the chat somewhat helpful and 4.07 times more likely to respond to the chat helpfulness question (question 2) with a “Yes” than those who did not seek COVID-19 information. Users seeking an in-person appointment were approximately 2.46 times more likely to find the chat somewhat helpful and 1.99 times more likely to respond to the chat helpfulness question (question 2) with a “Yes” than those who did not seek an in-person appointment. Users seeking telehealth services were approximately 2.48 times more likely to find the chat “Somewhat” helpful and 1.9 times more likely to respond to the chat helpfulness question (question 2) with a “Yes” than those who did not seek telehealth services. Users seeking vaccination were approximately 2.9 times more likely to find the chat “Somewhat” helpful and 3.85 times more likely to respond to the chat helpfulness question (question 2) with a “Yes” than those who did not seek COVID-19 vaccination.

We can conclude that our second hypothesis is supported regarding the fact that providing an actionable solution to chatbot users positively increases user perceptions of chat helpfulness.

## Discussion

### Principal Findings

The primary contribution of this study was to identify two main factors for designing chatbots for increased helpfulness: (1) identification of a target user group, in this case, a higher-risk user population for COVID-19; and (2) providing relevant actionable items for the target population. Extending access to care while also limiting patient and provider exposure to COVID-19 was a core motivation for implementing the Prisma Health chatbot. This concept is supported by previous research, with one early study reporting that telehealth availability expands access to care [70]. That same study indicated that telehealth users were younger and healthier than those who visited physicians’ offices or the emergency department for similar conditions. Our study results showed that chatbot users who believed themselves to be at risk of COVID-19, those with comorbidities, those aged >65 years, those exposed to other people with the virus, and those reporting COVID-19 symptoms found the chatbot service more helpful than those who may be younger, healthier, or at a lower risk of having the disease.

The results also showed that users who engaged with a relevant actionable item as a result of the chatbot conversation found the chat more helpful. The actionable items selected at the end of the chat conversation may have increased user perceptions of the helpfulness of the chat by assisting the user in taking meaningful action after better understanding their COVID-19 condition.

Helpfulness can be considered a component of a larger grouping of constructs aimed at assessing system effectiveness, utility, or beneficence and can be a key measure of program quality and a direct indicator of satisfaction [71] and overall success. Thus, these results may indicate that the chatbot in this study helps the health care organization achieve its objective of providing helpful technology-enabled engagement for people who are at higher risk of having COVID-19. The results suggest that the chatbot in this study was helpful for the health care organization to identify the higher-risk target audience as well as provide them with an actionable item resulting from the conversation with the chatbot. Additional studies are needed to generalize the findings to the broader context of health care systems and chatbot experiences.

An important purpose of the chatbot from an organizational perspective was to take advantage of accessible, lower-cost, virtual visit technologies. As noted in a recent study, telehealth visit costs are lower than in-person alternatives at retail health clinics, urgent care centers, emergency departments, and primary care physician offices for acute, nonurgent conditions [72]. In contrast, direct-to-consumer telehealth may increase access by making care more convenient for certain users, but it may also increase use and health care spending [73]. In line with this previous study, the chatbot in our study was designed to provide patients and the general public in the geographic region with a low-cost, easy-to-use, nonintrusive entry into the health care system before using a video-based telehealth option. The long-term downstream cost impacts of chatbots should continue to be studied [74].

There are several limitations to this study. We used convenience sampling in our methods by conducting a secondary analysis of real-world observational chatbot data, thus lacking sociogeographic and demographic controls. We do not know if the same results will be true of chatbot users in other locations or with conditions outside this case study and outside the COVID-19 pandemic context. The observational data lacked a deeper understanding of the users, including their socioeconomic status, educational level, ethnicity, and other data points common in purposively controlled studies. We also could not determine whether the users were using the chatbot for themselves or as caregivers. The question used in this study to assess helpfulness contained a positive-leaning bias. The question and response options were kept simple to maintain ease of use for users. However, the assessment of the results should take this into account. Furthermore, the symptoms were purely self-reported in the chatbot, with no way to clinically validate the symptom responses before a provider interaction. Users were people from 2 major regions of 1 state, all of whom sought information from the website of a single health care enterprise. Thus, only people with a computing device and internet access were able to use the system. The chatbot used in this study was offered in both English and Spanish. No other languages were supported. There were too few responses in Spanish to conduct a reliable analysis.

Further research is needed to understand individuals’ expectations, needs, perceptions, and experiences relative to the use of AI chatbots, especially by certain demographics such as older age groups. Qualitative studies could be useful in this regard, for example, to understand what factors lead a user to abandon the chat and to further understand the wide range of missing chat response data. Further research is needed to understand the effects that the use of chatbot technologies will have on health care use. Future research should assess chatbot satisfaction across a range of measures beyond helpfulness, such as trustworthiness, by assessing a wider range of user demographics beyond age.

### Conclusions

Users at higher risk of COVID-19 found the chatbot technology in this study to be more helpful, indicating that the use of remote patient engagement tools offered by the local health care system may provide value to the local community as an important response to the pandemic. In addition, this study demonstrated that many users intend to engage further with the health care system beyond their chatbot experience to schedule virtual visits, speak with a COVID-19 consultant over the phone, and schedule an appointment with a local provider. These findings may aid health care systems in their current and future chatbot implementations for COVID-19 or for other conditions that greatly affect the operations of health care systems. The helpfulness of interactive technology is an important way to measure the overall effectiveness of a conversational recommender system and strategy. We conclude that users of the system engaged in a manner that seemed to be focused on their personal well-being and that the virtual care strategy used was designed to engage patients and the public and to manage scarce resources as effectively as possible. We also conclude that chatbot design considerations should focus on using just those features that are most beneficial to the target audience and affected providers. Thus, identifying high-impact features, conversational pathways, and recommendations can be very helpful to inform the design of future conversational recommender systems or chatbots.

## Appendix

Multimedia Appendix 1 Mapping for activity categories and chatbot activity links.

## Abbreviations

AI: artificial intelligence
aOR: adjusted odds ratio
CDC: Centers for Disease Control and Prevention
OR: odds ratio
