# Supplementary material for: Effects of User-Reported Risk Factors and Follow-Up Care Activities on Satisfaction With a COVID-19 Chatbot: Cross-Sectional Study
Source: JMIR Mhealth Uhealth. 2023 Dec 14;11:e43105. doi: 10.2196/43105 (PMC10727483; doi:10.2196/43105)
Supplement: Multimedia Appendix 1 [file mhealth_v11i1e43105_app1.docx]

Multimedia Appendix 1

Table 1: Mapping of Activity links presented in chatbot to activity groups.

| **Activity Group** | **Coding Value** | **Activity Link in Chatbot** |
| --- | --- | --- |
| Seeking COVID-19 Info **(A1)** | (1) | Clicked link: https://www.prismahealth.org/coronavirus/ |
|  | (0) | Missing (System) |
|  |  | Total |
| Contact PRISMA **(A2)** | (1) | Patient clicked phone number -1-844-447-3627 |
|  | (0) | Missing (System) |
|  |  | Total |
| Seeking In Person Appointment (**A3)** | (1) | Clicked link: https://doctors.thephqc.org/search?primary_care=Primary%20Care%20Providers&sort=relevance%2Cnetworks  OR  Clicked link: https://doctors.myhfn.org/search?primary_care=Primary%20Care%20Providers&sort=relevance%2Cnetworks  OR  Clicked link: https://www.healthgrades.com/usearch?what=coronavirus  OR  Clicked link: https://doctors.prismahealth.org/search?sort=relevance&unified=primary%20care&filter=region%3AUpstate  OR  Clicked link: https://doctors.thephqc.org/search |
|  | (0) | Missing (System) |
|  |  | Total |
| Seeking Telehealth Appointment **(A4)** | (1) | Clicked link: <https://www.prismahealth.org/virtual-visit>  OR  Clicked link: https://prismahealth.org/services/virtual-care |
|  | (0) | Missing (System) |
|  |  | Total |
| Seeking Vaccine (**A5)** | (1) | Patient clicked phone number -1-833-277-4762  OR  Clicked link: https://lp.prismahealth.org/prisma-health-vaccination-sites/ |
|  | (0) | Missing (System) |
|  |  | Total |
| Seeking Travel Guidelines **(A6)** | (1) | Clicked link: <https://travel.state.gov/content/travel/en/traveladvisories/traveladvisories.html/>  OR  Clicked link: https://www.cdc.gov/coronavirus/2019-ncov/travelers/ |
|  | (0) | Missing (System) |
|  |  | Total |
| Seeking Vaccine Info **(A7)** | (1) | Clicked link: <https://www.cdc.gov/coronavirus/2019-ncov/index.html>  OR  Clicked link: https://scdhec.gov/covid19/covid-19-vaccine  OR  Clicked link: https://www.prismahealth.org/coronavirus/covid-19-vaccine |
|  | (0) | Missing (System) |
|  |  | Total |
